# Precision cut lung slices: an integrated ex vivo model for studying lung physiology, pharmacology, disease pathogenesis and drug discovery

**DOI:** 10.1186/s12931-024-02855-6

**Published:** 2024-06-01

**Authors:** Cynthia Koziol-White, Eric Gebski, Gaoyaun Cao, Reynold A. Panettieri

**Affiliations:** Rutgers Institute for Translational Medicine and Science, The State University of NJ, 08901 Rutgers, New Brunswick, NJ USA

**Keywords:** Precision cut lung slices, Respiratory therapeutics, Airway physiology in precision cut lung slices

## Abstract

Precision Cut Lung Slices (PCLS) have emerged as a sophisticated and physiologically relevant ex vivo model for studying the intricacies of lung diseases, including fibrosis, injury, repair, and host defense mechanisms. This innovative methodology presents a unique opportunity to bridge the gap between traditional in vitro cell cultures and in vivo animal models, offering researchers a more accurate representation of the intricate microenvironment of the lung. PCLS require the precise sectioning of lung tissue to maintain its structural and functional integrity. These thin slices serve as invaluable tools for various research endeavors, particularly in the realm of airway diseases. By providing a controlled microenvironment, precision-cut lung slices empower researchers to dissect and comprehend the multifaceted interactions and responses within lung tissue, thereby advancing our understanding of pulmonary pathophysiology.

## Introduction

**Fig. 1:**
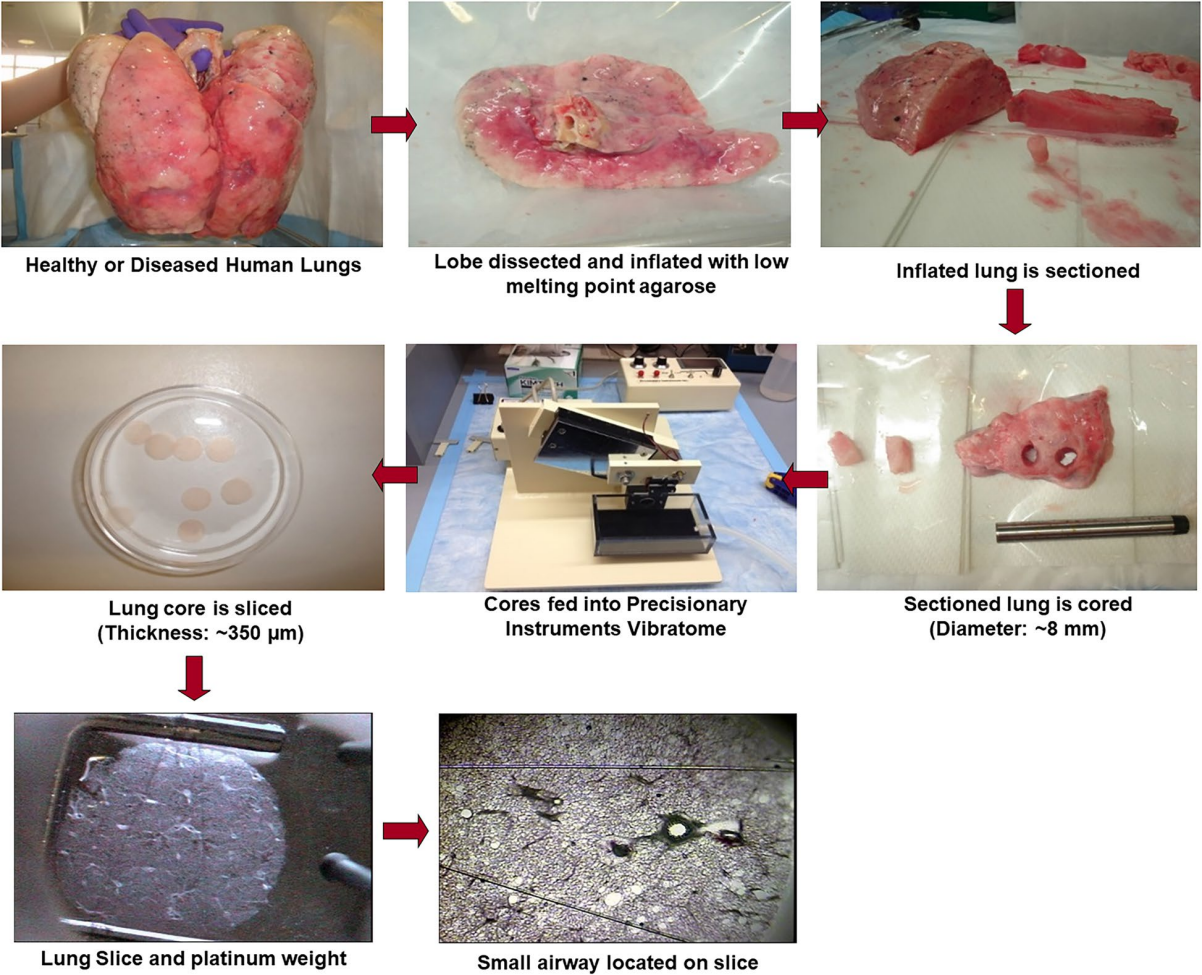
Generation of human lung slices. The lobes of the lungs from either healthy or diseased donors were inflated with a low melting point agarose, the agarose was allowed to solidify in the lungs, and the lung tissue was sectioned. A tissue punch or coring tool was used to generate columns of tissue containing airways, which are fed into a Precisionary Instruments Vibratome© to generate ~350 µm thick slices. Airways in these slices were identified, and the slices were weighed by platinum weights with nylon threads to assess changes in contraction and relaxation of the airways

The history of precision-cut lung slices can be traced back to the general development of techniques for preparing and studying tissue slices in general. The development of tissue-slicing techniques began in the late 19th and early 20th centuries when investigators used specialized instruments to cut thin sections of various tissues for microscopic examination, but the use of the microtome for preparation of lung slices was first published in 1944 [1]. The term “precision cut” implies a high degree of accuracy and consistency in the preparation of tissue slices. In the mid-20th century, advances in microtome/vibratome technology and other cutting instruments allowed for the creation of slices with more precision, as depicted in Fig. 1. The application of precision-cut techniques to lung tissue likely followed the general trends in tissue slicing.

Initially, lung slices were employed for toxicology studies to examine cellular survival in the face of exposure to environmental/industrial pollutants [2–5]. Subsequently, the importance of maintaining the physiological composition of the tissue for experiments was recognized, and precision-cut lung slices gained popularity and acceptance (as reviewed in [6]). Concerning respiratory research, PCLS became particularly valuable for studying airway reactivity and lung function, fibrosis, vascular responsiveness, responses to pharmacological agents/therapeutics, and airway immunology. The PCLS platform maintains the architecture of the lung tissue, including the airways, blood vessels, and parenchyma with the study of resident cell types in the context of the whole lung tissue. Over the years, advancements in imaging technologies and tissue preparation methods helped to improve the quality, rigor, and reproducibility of studies conducted using lung slices. Researchers can now study dynamic processes in real-time, such as airway constriction and dilation with greater accuracy, and can expose the slices to various substances to assess their effects on lung function and selective cellular responses, all of which provide insight into drug development and safety evaluation of potential therapeutic. PCLS provides a more realistic simulation of the lung microenvironment to study a variety of lung diseases.

**Fig. 2:**
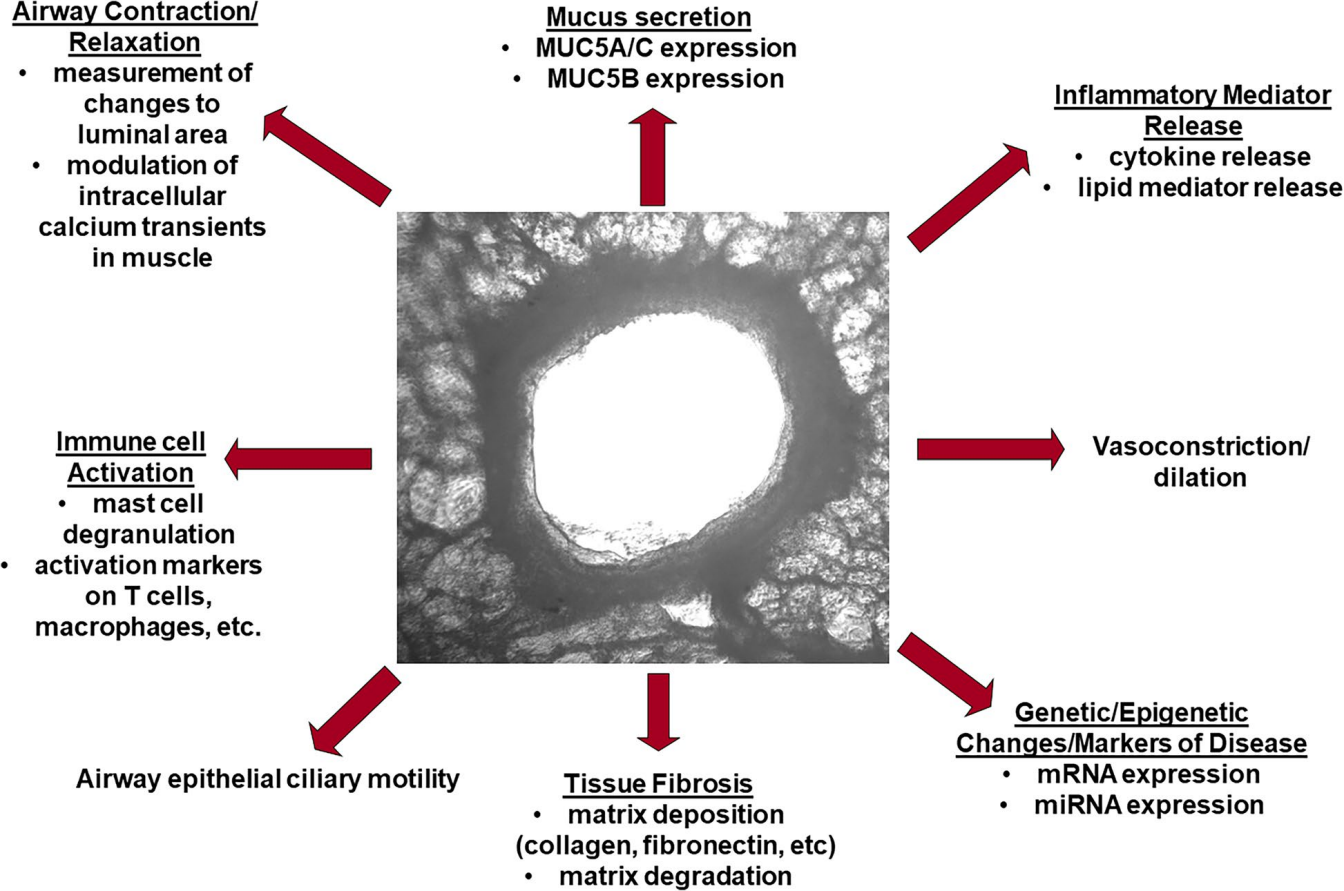
The range of processes that can be measured utilizing the PCLS as a model system. A wide variety of endpoints, from changes in gene expression to tissue fibrosis and more, can be studied using this model system. Specific outcomes are noted below each type of outcome measured

Taken together, PCLS have emerged as a powerful tool to study an array of lung diseases, including asthma, chronic obstructive pulmonary disease, fibrotic lung diseases (idiopathic pulmonary fibrosis, sarcoidosis), diseases of the pulmonary vasculature (pulmonary arterial hypertension and bronchopulmonary dysplasia), acute respiratory distress syndrome, lung cancer, and the consequences of respiratory pathogen exposure. The functional complexity, physiological relevance, and versatility of the platform make PCLS an invaluable asset for the study of the complexities of lung diseases and for advancing therapeutic development. As researchers refine the generation and uses of PCLS, the platform is poised to play a pivotal role in deepening our understanding of lung diseases and ultimately improving clinical outcomes for patients with those diseases. This review will summarize the use of PCLS in examining various lung diseases, and how this platform can foster an understanding of fundamental aspects of lung biology and disease-specific pathobiology, as depicted in Fig. 2. Further, the platform can serve as a tool for novel therapeutic discoveries in lung diseases.

### Studies in infectious disease

Respiratory pathogen exposure has been extensively studied in primary and immortalized airway cell types; PCLS, however, offers an integrated tissue model to study the consequences of exposure and infection. Responses of the tissue to a variety of respiratory pathogens have been examined including responses to influenza, rhinovirus, respiratory syncytial virus, SARS-CoV2, fungal lung infection, and pathogenic bacterial species like *Pseudomonas aeruginosa* and *Mycobacterium tuberculosis*.

An influenza study used PCLS to show that exposure of PCLS downregulated albumin uptake, which impairs protein clearance from the alveolar space during flu-induced acute respiratory distress syndrome [7], and that the combination of influenza A and cigarette smoke exposure diminished responses to bronchodilators used in the treatment of asthma and COPD [8]. Activation of virus-sensing pathways induced by influenza and its competency for replication in PCLS following cigarette or e-cigarette exposure provided the model to report that cigarette exposure of the lung tissue worsened responses to influenza by suppressing the ability of the lung to properly respond to infection [9, 10]. Single cell sequencing of cells derived from PCLS showed that vaping extract amplified influenza-induced inflammatory responses [11]. Studies also examined the effects of specific inhibitors of multiple pathways to treat influenza infection and its effects [12–15], as well as to provide information on fundamental ways in which the lung responds to influenza infection [16–20]. Exposure to some species-specific viruses, including canine distemper virus [21] and murine pneumonia virus [22], have also been examined using PCLS.

The functional consequences of respiratory viruses like rhinovirus (RV) and respiratory syncytial virus (RSV), both of which evoke the development of wheeze and asthma as well as induce asthma exacerbations, have leveraged the use of PCLS. Lung inflammation following exposure to RSV in PCLS [23] identified novel treatment strategies including mucosal application of an RSV vaccine [24] and modulation of an ion channel [25] to attenuate the effects of RSV. Much of the research that has been conducted examining the effects of RV on the airways has focused on examining inflammatory mediator release from monocultures of airway epithelial cells. Interestingly, RV exposure of PCLS can modulate both agonist-induced contraction [26, 27] and relaxation [28] of the airways that both appear to be serotype- and disease state-specific. Inflammatory responses elicited by RV have also been studied in PCLS [29], with the addition of different treatment strategies showing a reduction in RV-induced inflammation [30, 31].

With the SARS-CoV2 pandemic catapulting respiratory virus exposure to the forefront of research efforts for scientists globally, PCLS provided a relevant platform to study aspects of infection and response to infection in the lung. An early study utilized various strains of infectious bronchitis virus, which are avian coronaviruses, to examine infection of the lung with these viruses [32]. An understanding of the mechanisms driving the inflammation associated with SARS-CoV2, as well as replication mechanisms and pathologic features of the disease have been achieved using PCLS [33–35]. Other laboratories identified effective therapeutics that could target SARS-CoV2 infection in PCLS [36–40].

While there has been extensive focus on exposure of lungs to viral pathogens, there are also pathogenic bacteria and fungi that infect the lung causing inflammation and disease. Investigators have used components of bacteria, including lipopolysaccharide (LPS), to stimulate pattern recognition receptors on a variety of cell types in PCLS. LPS induced an upregulation of innate immune responses consistent with endotoxin challenge of patients [41]; LPS had little effect on agonist-induced airway constriction or relaxation [42]; and toll-like receptor 2 (TLR2) activation reduced the ability of bronchodilators to induce relaxation of murine airways [43]. The consequences of exposure to the pathogenic bacteria *Pseudomonas aeruginosa* has also been examined in PCLS [44–47], with one study showing that some commensal bacteria strains present in the lung of cystic fibrosis patients may protect the host from *P. aeruginosa*-induced inflammation [46], and another noting a more robust immune/inflammatory response in younger mice compared to older mice of the same strain when exposed to the bacteria [47]. Some bacteria, like *Trueperella pyogenes*, only appear to infect animals but not humans, with their pathogenic effects having been studied in PCLS [48]. Additional studies in PCLS studied *Mycobacterium tuberculosis* [49], *Mycobacterium abscessus* [50], *Yersinia pestis* [51], and *Staphylococcus aureus* [52] to understand the pathologic features of infection and agents used to treat these infections. Additionally, co-infection of PCLS with influenza and a *Mycobacterium* strain showed that the influenza infection increased the susceptibility to *Mycobacterium* infection by attenuating responses to the bacteria that would otherwise allow the animal to clear the bacteria [53]. Exposure of the lung to fungal pathogens can also occur in healthy patients but tend to be a significant problem for those with underlying lung diseases. Infection of murine PCLS with *Pneumocystis murina*, a common pathogen that infects immunosuppressed mice and can cause pneumonia, showed colonization of PCLS with the pathogen and that PCLS can serve as a useful model for testing anti-fungal molecules in a moderate-to-high throughput manner [54]. Overall, PCLS can serve as a valuable model to study the pathologic and inflammatory aspects of exposure to bacterial and fungal pathogens, as well as aid in the discovery of novel, effective treatments to combat these pathogens.

### Studies in lung cancer

Although considerable research has been done to study lung cancer and both current and potential therapeutics, few studies have used PCLS as a model system. Some studies have utilized lung cancer explants into murine systems and analyzed the PCLS derived from these mice, where others have used cancerous tissue derived from the lungs of patients to generate PCLS. A few studies have utilized PCLS as a preclinical model to test therapeutics in both human and mouse tissues [55–58]. Others have examined lymphocyte migration into lung tumors to try to understand lymphocyte accumulation in the tumors [59], while another study examined changes in macrophage activation and the contribution to lung cancer growth [60]. A proof-of-concept study leveraged molecular imaging of PCLS from non-small cell lung cancer tissue to examine cell-cell and cell-stroma interactions in lung cancer [61]. There have also been studies targeting specific signaling pathways driving some lung cancers that have provided insight into the utility of specifically targeting the lung to treat the cancer [62–68]. Collectively, PCLS use in cancer studies is still evolving, but the model is amenable to moderate to high-throughput drug discovery for cell-targeted therapy to the lungs.

### Studies in pulmonary vascular diseases

According to the National Institutes of Health, pulmonary hypertension (PH) has an incidence rate of about 1% globally. However, in over 50% of the cases of PH there is no known cause. PCLS have been used to study vasoconstriction and dilation [69], and to model the consequences of exposures that induce bronchopulmonary dysplasia (BPD) and acute respiratory distress syndrome (ARDS). Studies of endogenous vasoconstrictors and dilators have used PCLS [70–75], along with studies examining the consequences of pharmacologic intervention on these processes [76–83]. Two studies also noted that cigarette smoke enhanced the contraction of vessels in PCLS to endothelin-1 [84, 85], an endogenous vasoconstrictor. One study examined a role for IL-11 in PH, finding that in PH patients IL-11 expression was higher in the vasculature from those patients compared to patients without PH, and that IL-11 treatment of PCLS made the vessels more sensitive to endothelin-1-induced vasoconstriction [86]. Changes elicited by exposure to a hypoxic [87–95] or hypercapnic [96] environment, both of which can cause increased vascular resistance, have been modeled in PCLS. For BPD, the hyperoxia and mechanical ventilation that are necessary for ventilation of premature newborn lungs causes damage to the lungs that can persist long-term. Few studies exist using PCLS to examine mechanisms of pathology of BPD [93, 97–99] or ARDS [100, 101] and even fewer have studied mechanisms of pulmonary hypertension [102, 103]. Despite a lack of extensive studies, PCLS may provide insight into the pathophysiology and discovery of new therapeutic approaches in the treatment of pulmonary vascular diseases.

### Studies in fibrotic lung diseases

Idiopathic pulmonary fibrosis (IPF) is a fibrotic disease of the lung tissue surrounding the alveoli that progressively stiffens the lung, making it difficult for the person to breathe. Studies in PCLS have supported a number of different molecules in the pathogenesis of IPF including: a transmembrane protein that can interact with growth factor receptors or extracellular ligands to modulate receptor activation [104]; activation of histone deacetylases [105]; activation of integrins [106]; ion channel activation [107]; a kinase and a signaling microdomain protein [108]; a protein involved in cell fate determination, motility, and organogenesis [109]; and even an miRNA mimic as a potential therapy [110]. Additionally, other pathways have been postulated to be part of specific aspects of the biology of both epithelial cells and fibroblasts that may play a role in the pathology of IPF [111–121]. Studies have utilized PCLS to identify the cell types that drive fibrosis signals and showed the ways in which PCLS can model IPF [122–125]. A number of pharmacologic inhibitors have been used as potential treatments for IPF [126–131], including current therapeutics that are being used in the treatment of IPF, like nintedanib and pirfenidone [132–134]. The contribution of released factors to the development/progression of IPF has also been studied in PCLS [121, 135, 136]. PCLS provides an ex vivo system in which lung tissue derived from IPF patients can be examined for biomarkers, and to ascertain the efficacy of a variety of therapeutic interventions to decrease expression/deposition of the fibrotic proteins that are overproduced in the disease that contribute to the increased stiffness of the lungs.

Sarcoidosis is a systemic inflammatory disease that affects multiple organs of the body. Broadly, sarcoidosis induces granuloma formation in the tissue and in the lungs produces a fibrosis-like phenotype that, like IPF, induces severe, irreversible damage to the lungs. To date, there are no studies examining PCLS derived from the lungs of sarcoidosis patients. PCLS may provide a platform well suited for study of the fibrosis associated with sarcoidosis, allowing for testing of novel therapeutics and identification of biomarkers similarly to how IPF is being studied in PCLS.

### Studies of obstructive lung diseases

PCLS models have been used to study the consequences of obstructive lung diseases including asthma and chronic obstructive pulmonary disease (COPD). A study showed that airway constriction and relaxation in PCLS linked internal perimeter of the airways to airway smooth muscle shortening [137]. Additionally, PCLS has enabled investigators to translate their findings to clinically measurable respiratory parameters that are typically measured in obstructive lung diseases like asthma and COPD. For example, the luminal area of the small airways in PCLS correlates with the forced expiratory flow between 25% and 75% (FEF25-75), which serves to predict small airways obstruction that is characteristic of asthma and COPD (as reviewed in [138]).

With respect to allergic asthma, studies in both human PCLS and in animal models of allergic airways inflammation have enhanced our understanding of basic mechanisms underlying bronchoconstriction and how an inflammatory milieu affects basal bronchomotor tone. Early studies using human PCLS (hPCLS) demonstrated that allergen sensitization of hPCLS, and subsequent stimulation with allergen, could mimic bronchoconstriction associated with allergic asthma [139, 140]. Since then, human- [141–144], rat- [145], and guinea pig-derived [146, 147] PCLS have been utilized to determine the roles of specific receptor subtypes and signaling molecules downstream of the immunoglobulin E (IgE) receptor in IgE-induced airway constriction. Murine models of allergen exposure, or exposure to proteases found in allergen extracts, showed that release of specific inflammatory mediators [148] increased airway contractility [148–151]. In the context of a Th2 inflammation of the airways, compelling evidence suggests that Interleukin-13 (IL-13) and IL-4, Th2 cytokines, enhanced airway contractility and diminished responsiveness to commonly used bronchodilators in human, rat, and murine PCLS [141, 152–156]. In animal models of allergic airway inflammation, PCLS studies revealed a spectrum of cellular pathways that evoke airway hyperresponsiveness including transcription factor activation in airway cells [157]; asthma-associated genes modulating airway smooth muscle shortening [158]; and increased cholinergic stimulation of nerve-dependent airway constriction following ovalbumin sensitization and challenge [159] following early-life allergen exposure [160]. Stimulation of PCLS with other inflammatory cytokines that are associated with allergic asthma, non-allergic asthma, and asthma exacerbations have been shown to alter contractility of the airways and/or attenuate agonist-induced bronchodilation (specific studies listed in Table 1) [141, 152, 155, 156, 161–165].

**Table 1:**
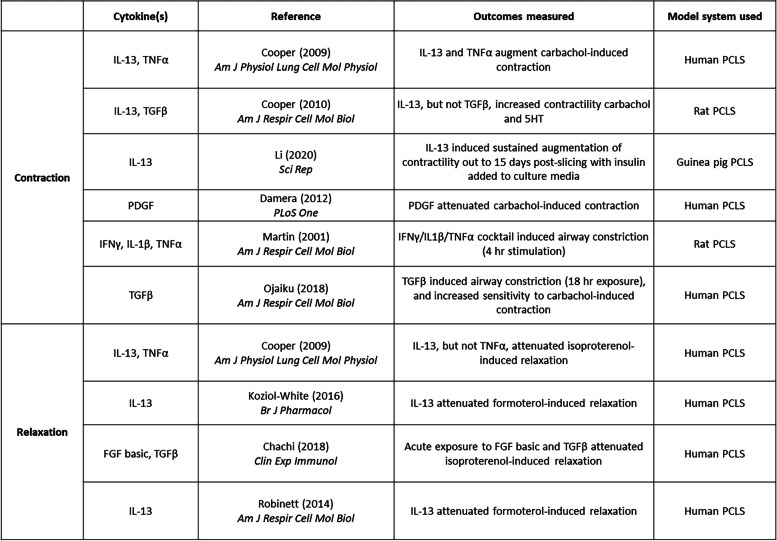
Studies of inflammatory mediator-dependent modulation of airway contraction/relaxation

To understand basic mechanisms of airway biology, PCLS can be used to examine mechanisms that underly contraction or relaxation in non-diseased tissue from either human or animal models. The role(s) for numerous signaling molecules in mechanisms of receptor-mediated contraction and relaxation of small airways have been examined by observing narrowing and opening of airways (specific studies listed in Table 2) [75, 151, 166–182], and/or visualization of signaling events occurring simultaneously with airway contraction/relaxation (specific studies listed in Table 3) [75, 158, 169, 170, 175, 183–196]. Airway constriction in PCLS also induced remodeling in guinea pig PCLS [197]. Bronchopulmonary dysplasia can alter the vasculature architecture of the lung, inducing susceptibility for the development of asthma. One study noted that exposure of mouse pups to hyperoxia, that induces a BPD-like phenotype, evoked greater maximal airway contraction in PCLS derived from the hyperoxic mice compared to those in normoxic conditions [198]. PCLS has also had value in modeling of clinically observed phenomena, including desensitization of the β_2_ adrenergic receptor (β_2_AR) following agonist stimulation [155, 199–202].

**Table 2:**
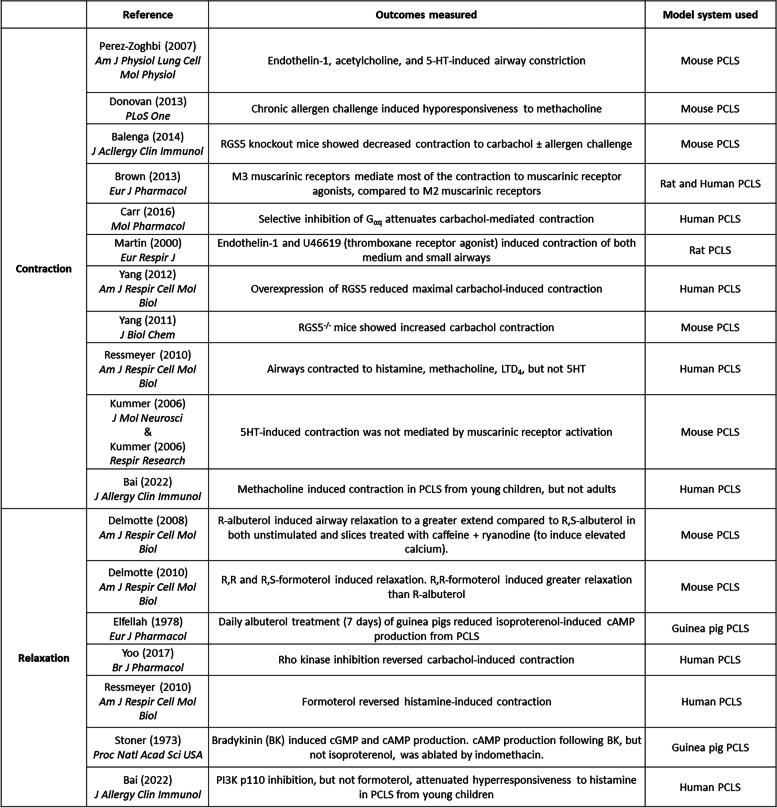
Studies of airway contraction/relaxation

**Table 3:**
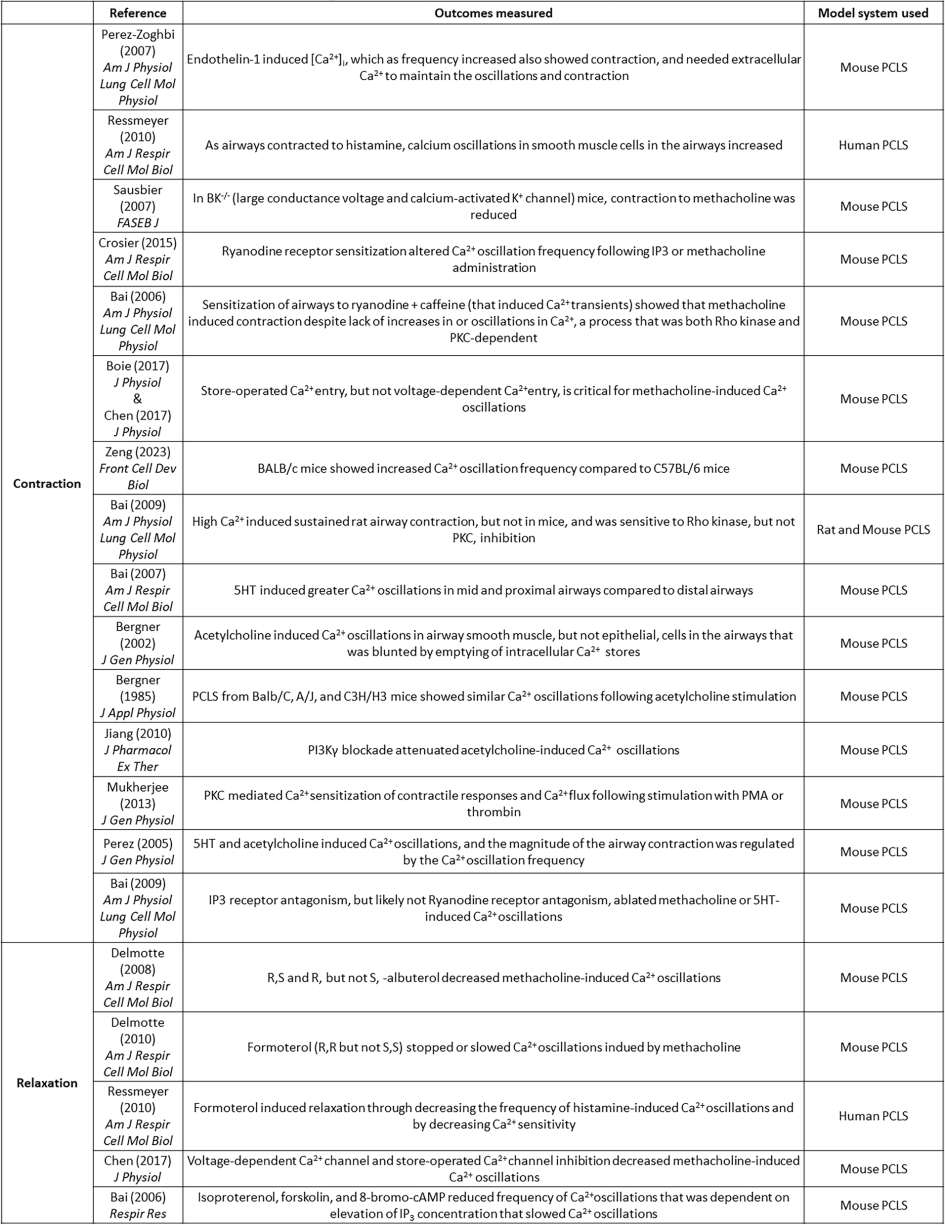
Studies showing visualization of processes associated with airway contraction/relaxation

Despite significant advances in our knowledge of asthma pathogenesis, the search for better bronchodilators is still evolving. Drugs that inhibit pathways underlying contraction promote relaxation of pre-constricted airways [150, 155, 162, 168, 178, 180, 203–207], and testing of selective molecules that either elicit or enhance bronchodilation have been used in PCLS [81, 208–212]. Other non-canonical pathways have also been targeted to reverse, or inhibit, airway contraction (specific studies listed in Table 4) [155, 165, 202, 213–228]. Compounds targeting orphan, or non-β_2_ receptors, can serve as new classes of bronchodilators, or have been suggested to be useful as add-on therapy for existing therapeutics [164, 165, 214–228]. Overall, PCLS provide a highly useful and versatile platform for drug discovery to modulate both contraction and relaxation of the airways that can serve to increase our understanding of basic mechanisms underlying these processes, and uncover novel therapeutics that may have clinical use in treatment of obstructive lung diseases.

**Table 4:**
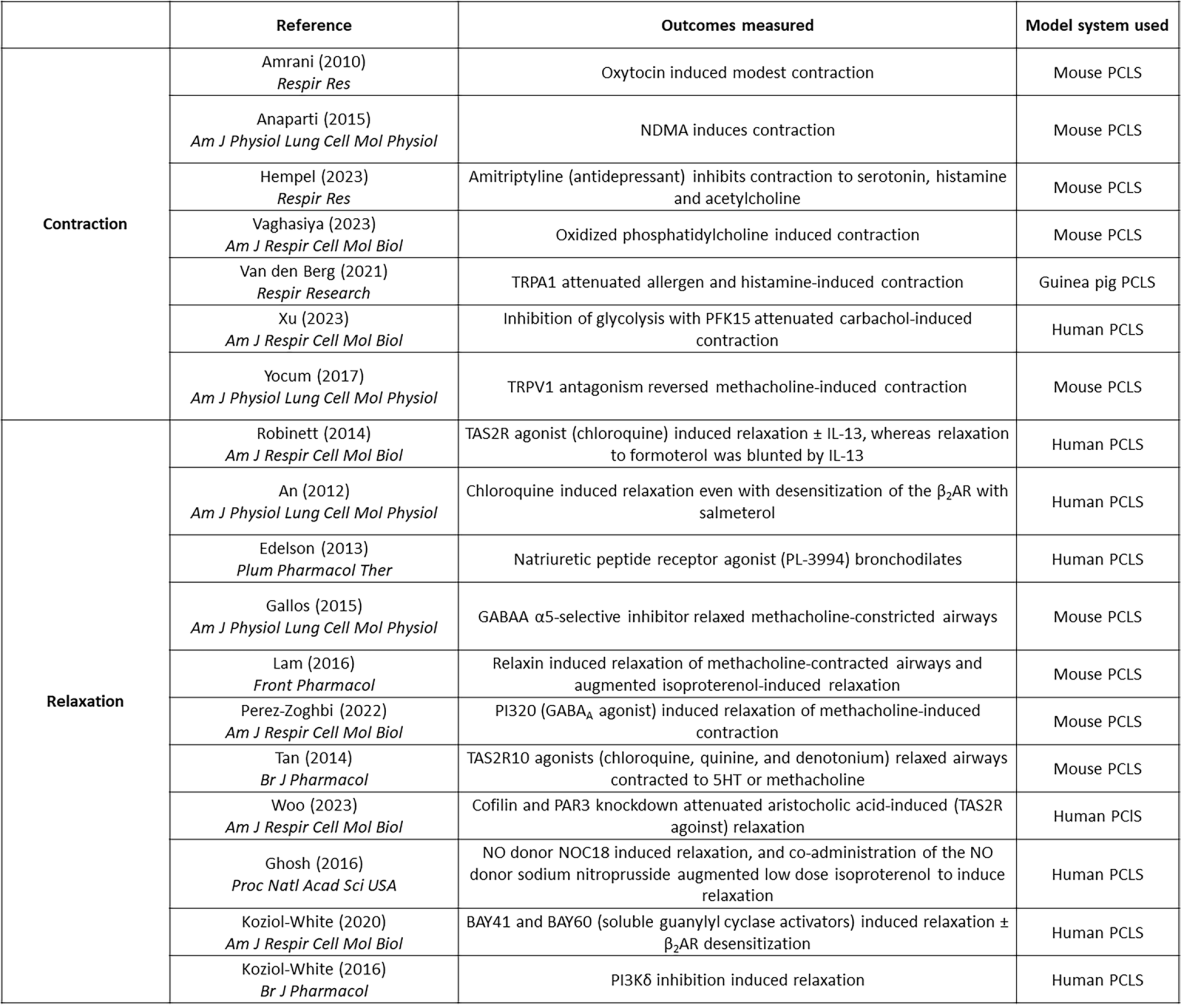
Studies of novel bronchoconstrictors and bronchodilators

Despite considerable research into modeling asthma phenotypes and/or inflammatory milieu associated with asthma, there is a paucity of research into chronic obstructive pulmonary disease (COPD) using PCLS. To date, only four studies examining aspects of COPD pathobiology in hPCLS have been published [229–233]. Van Dijk et al. reported that elastase-induced parenchymal disruption of murine PCLS that would provide an animal model to study COPD [234], and Kim et al. defined the mechanical properties of PCLS derived from lungs from an emphysema patient [235]. Ideally, PCLS derived from patients with COPD would identify biomarkers that could be targeted, thereby aiding in discovery of drugs that would mitigate the inflammation and destruction of the airspace associated with the disease.

### Environmental toxicant exposure studies

An early adaption of PCLS for research focused on PCLS use in lung toxicology [2–5]. Since then, an array of other endpoints have been studied to understand how exposure to various toxicants can engender inflammation and even promote airway hyperresponsiveness. Consequences of exposure to noxious gases, including warfare agents like sarin and VX gas, in PCLS showed enhanced airway constriction that could be modulated by anti-muscarinic drugs [236–239]. Chlorine exposure of PCLS increased release of inflammatory mediators from the tissue, decreased airway constriction, and decreased cell viability [240, 241]. Exposure to gases from industrial processes or the use of pesticides induced cytotoxicity in PCLS, as well as inflammatory mediator production and oxidative stress [242, 243]. Interestingly, lipid peroxidation occurs in many disease processes and occurs following toxicant exposure. Recently, a PCLS study detected lipid peroxidation using a biosensor following exposure to nitrogen mustard [244]. Such a biosensor can provide a tool for understanding of toxicity of a variety of environmental compounds. Additionally, another study found that the effects of nitrogen mustard exposure that drive pulmonary toxicity appear to be independent of immune cell trafficking to the lungs [245].

In addition to noxious gases, exposure to environmental cigarette smoke (CS), has been studied in PCLS. CS or CSE (cigarette smoke extract) elicited inflammatory mediator release, induced histologic inflammatory changes and extracellular matrix gene expression, decreased cell viability, increased markers of the unfolded protein response, and increased airway constriction to serotonin but not methacholine [246–249]. Interestingly, menthol-containing e-cigarette condensate decreased airway contraction in PCLS but increased oxidative stress markers [250]. The combination of influenza with CS exposure showed that CS exposure reduced flu-induced inflammatory mediator release, and the combination of the two insults reduced airway relaxation when CS exposure alone had little effect [8]. Using a highly sensitive sensor for cAMP, investigators showed that CS exposure of mouse PCLS attenuated β_2_AR signaling that was reversed by inhibition of phosphodiesterase 3 (PDE3) and PDE4 [251].

The effects of chemical sensitizers, whether inhaled or dermal sensitizers, have been studied in PCLS where most sensitizers induced inflammatory mediator release from PCLS [252, 253], but only a few increased contractility of the airways [252, 254, 255]. Additionally, the toxicologic effects of various drugs and chemicals have been assessed in PCLS. Exposure of PCLS to an anti-cancer drug that is known to cause pulmonary toxicity showed increased inflammatory mediatory release as well as cellular toxicity [256]. PCLS exposed to the industrial toxins cadmium chloride, ammonium hexachloroplatinate, and zinc chloride showed increased inflammatory mediator release from PCLS [257–259], and when cadmium chloride was combined with Transforming Growth Factor β (TGFβ) there was increased fibrosis of the tissue compared to TGFβ treatment alone [257].

Common environmental exposures that induce a significant number of exacerbations of underlying asthma, including ozone and particulate matter, have also been studied using PCLS. Following exposure to ozone, inflammatory markers were upregulated in PCLS [260–262] and acute exposure to high levels of ozone also induced airway hyperresponsiveness in the absence of influx of immune cells [260]. Interestingly, deletion of microsomal prostaglandin E synthase 1 (PGES-1), an enzyme necessary for the metabolism of arachidonic acid into prostaglandin E2, had little effect on ozone-induced airway hyperresponsiveness and inflammation in a mouse model [262]. Diesel exhaust particle exposure of PCLS induced cell death, oxidative stress, and inflammatory mediator release [263, 264]. Particulate matter, from agricultural dust or the desert, increased airway hyperresponsiveness [265], anti-oxidant gene expression, and inflammatory mediator gene expression [266] in PCLS.

### Comparisons of animal versus human models

Animal models provide platforms to study pathophysiology of human disease. Studies of lung diseases have extensively used rodent models, including mice and rats, and many studies have used PCLS derived from these animals. Only a few studies have compared animal-derived to human-derived PCLS. Schleputz et al. performed a study of PCLS from mice, rats, guinea pigs, marmosets, sheep, and humans found that electric field stimulation of neural responses that induced airway constriction in marmosets and guinea pigs was similar to humans, whereas the airways of mice and rats did not respond similarly to human [267]. Zeng et al. noted differences among strains of mice in their responsiveness to methacholine, a muscarinic receptor agonist, and 5-hydroxytryptamine (5-HT), a serotonin receptor agonist, where airway constriction of the small airways (not the tracheas) in PCLS of BALB/c mice to both contractile agonists elicited greater contraction compared to small airways from C57BL/6 [187]. Comparison between human and guinea pig-derived PCLS noted that airways from both contracted to leukotriene D4, thromboxane, histamine and methacholine, but only guinea pig airways contracted to serotonin [268]. A comparison among PCLS from a primate species and humans found that airways from cynomolgus macaques and baboons responded most similarly to human airways with respect to their responsiveness to methacholine, histamine, serotonin, leukotriene D_4_ (LTD_4_), and endothelin-1 [269].

In addition to differential responsiveness to bronchoconstrictors, many studies highlight the substantial differences between mice and human lungs in terms of the physiology, anatomy, and immunology of each species. Studies have also highlighted that the preclinical results obtained from mouse studies failed to accurately model airways diseases in humans (as reviewed in [138]). Despite the limitations of mouse models in the study of airways diseases, rodent strains offer the opportunity to genetically modify the animal to assess the function of cell-specific gene deficiency on the development and progression of lung diseases. Two studies have utilized siRNA-mediated knockdown of specific transcripts in human PCLS [225, 270, 271], but the siRNA used in each study was not cell-type specific. Despite the limitations of working with animal model systems, these platforms can augment our understanding of disease pathogenesis when combined with observations using human cells and tissues.

### Technologic innovations in PCLS use

With any model system, the broad utility and versatility of the system is what propels it into use on a greater scale, ultimately leading to significant use of the system. PCLS is becoming a platform that demonstrates both broad utility and versatility. Given this, studies by Martin et al. [81, 163, 172, 203, 267, 272, 273] and Sanderson et al. [158, 169, 170, 183–192, 195, 196] paved the way for technologic advances for studying obstructive lung diseases in PCLS with respect to imaging of airway and vascular constriction, but also visualization of cellular processes that underly airway constriction, like calcium oscillations in muscle surrounding the airways. Others have studied the effects of stretch of the tissue that mimics breathing in human lung, examining both mechanical and biological outcomes [274–280]. Utilization of PCLS to generate large data sets, like miRNA and RNAseq data sets, have also been optimized by a few laboratories [281, 282]. To understand how histone modifications alter inflammation of the lung, the histone acetyltransferase inhibitor MG149 was found to attenuate LPS and Interferon γ (IFNγ)-stimulated proinflammatory gene expression [283]. Others have also optimized siRNA-mediated knockdown of multiple gene targets to decrease protein expression [225, 270, 271], with one of the studies also assessing the functional effects of the knockdown [225]. Receptor localization has also been studied in the epithelium of the airways by fluorescent microscopy following adenoviral transduction of hPCLS following desensitization of the β_2_AR [199]. Investigators have imaged PCLS to visualize migration of live immune cells [284], to define 3D cell-cell interactions in situ [285], and to generate immune responses normally associated with immune responses observed to antigens that the individual has been vaccinated against [286]. Studies have also used electric field stimulation of PCLS and measured airway contraction [273], or exposure to specific wavelengths of light to induce relaxation of muscle in the airways [287]. PCLS have been utilized to examine mucociliary properties of airway epithelial cells [288–292], and the ability of lung tissue to repair itself [293]. A variety of pathologic processes, as well as cellular changes, have been examined using PCLS using a wide array of technologies.

### Advantages and challenges of the system

PCLS maintain the native architecture of the lung, including a complement of resident cells like airway smooth muscle, epithelial cells, fibroblasts, and resident immunocytes. This architecture provides the study of physiologic processes including airway constriction and dilation, vasoconstriction and dilation, lung fibrosis, and epithelial ciliary function. In diseased tissues, researchers can examine the reversal of some of the pathologies associated with the disease given experimental interventions. PCLS affords physiologic relevance to the research, as it is an environment that closely mimics the in vivo conditions and retains the 3D structure of the lung, thereby providing meaningful insights into disease mechanisms. Additionally, this system fosters the study of a multitude of outcomes through the interplay among various cell types. PCLS serve as invaluable tool for drug screening and therapeutic development, allowing for testing of both the safety and efficacy of compounds/biologics/etc. to expedite the translation of promising candidates for translation from bench to bedside.

With all the advantages of the system, there are some disadvantages to using PCLS to study lung diseases. The issues with the system are the following: lack of circulation, tissue viability over time, and standardization of derivation/culture/use of the slices between laboratories. While there is a wide array of cell types present in the lung tissue, the consequences of the effects of therapeutic intervention outside the lung on the resident lung cells, or on cell types recruited to the lung, cannot be studied. Only in in vivo systems, like rodent and non-human primate models, are the systemic responses and their effects on the lungs able to be studied in isolated PCLS following a given exposure. For human lung tissue, this simply is not possible. Due to the nature of generation of PCLS, there exists the limitation that exposure to small molecules/pathogens/etc. is non-physiologic as the entire slice, and all cell types contained within the slice, is/are likely exposed to a given stimulus. The use of slices in air-liquid interface cultures and delivery of some types of stimuli into the lumen of the airway directly may mitigate some of the off-target or non-physiological effects of a given exposure. Viability of the tissue over time is also an issue, depending on the outcome(s) being measured. For some assays including measuring ciliary beat or acute progression of induced fibrosis, short-term measurements pose no challenges. However, for other assays like assessment of airway contraction and relaxation, the fidelity and reproducibility of PCLS measurements decreases over time that the slices have been in culture. Baseline viability that is irrespective of therapeutic intervention that may be used to try to reverse the effects of processes like fibrosis also presents a challenge for long-term study of those types of lung pathologies. With respect to the differences in baseline variability, the acceptance criteria for lungs from “healthy” donors that have no history chronic illness can vary between laboratories with respect to things like O_2_ saturation of the donor near time of death, number of days on a ventilator, etc. Some laboratories use non-diseased resections from donors that have lung cancer, which calls into question whether they can be directly compared with tissue/PCLS from people with no cancer diagnosis. Standardization of a basic set of parameters for acceptance of tissue from “healthy” donors may help in decreasing experimental variability between laboratories.

With respect to the issue of viability of PCLS, groups have examined various outcomes following cryopreservation of PCLS to provide a larger supply of PCLS that can be utilized for more than just short-term culture [259, 294–298]. Bailey et al. showed that embedding PCLS in hydrogel biomaterials can extend the viability of the slices in culture [299]. With respect to cryopreservation, the methods vary from lab to lab, and vary among species that the PCLS were derived from. Watson et al. reported that PCLS were susceptible to zinc chloride-induced damage after cryopreservation [259], suggesting significant variation between freshly prepared and cryopreserved PCLS. Undoubtedly, standardization of the cryopreservation method and fidelity of the data produced in thawed tissue slices become much more difficult to achieve and assess when there is a lack of reproducibility or standard operating procedures. While some outcomes, like inflammatory mediator release, may exhibit less variability before and after cryopreservation, other outcomes like contraction and relaxation of the airways are subject to high variability from donor to donor even before PCLS are cryopreserved, most notably in human PCLS. Despite some successes, significant work is needed assure that outcomes of cryopreserved PCLS mimic those of fresh PCLS.

Other issues concern standardization of experimental conditions among laboratories. Multiple methods-focused papers have been published detailing the processing of mainly murine [66, 300–303] and human lung tissue [258, 304], highlighting both the complexities of generating PCLS and noting that becoming adept at the process of generating the slices can ensure greater reproducibility of results. These and other publications also show the differences in how murine PCLS are generated versus human PCLS, and demonstrate that the method(s) of generation even the same type of lung source (i.e. human lung tissue) can vary greatly between laboratories. Be it lung inflation protocols, tissue slicing protocols, culture media, or culture protocols, there is wide variation between labs that makes comparisons between studies challenging (partially noted in [6, 305]). With respect to culture media, Patel et al. described differences in long-term PCLS culture in a few different media formulations, noting that given certain inflammatory stimulation (LPS or poly(I: C)) that the robustness of cytokine release varied between the air-liquid interface (ALI)-cultured PCLS and submerged cultures as to which culture environment elicited a greater response [305]. The antimicrobial agents used in culture of PCLS vary slightly between studies, but most investigators use penicillin/streptomycin in the presence or absence of an anti-fungal agent to protect their cultures. The components added to a base media, including things like fetal bovine serum, vary between groups and can vary dependent upon the outcome that will be measured or the cell type that the investigators are interested in studying. For example, some groups do not use any serum in their media formulation because there isn’t a need for growth of the structural cells in culture, but rather maintenance of the architecture of the tissue. For study of epithelial cells lining the airways, some groups have used epithelial cell-specific media that is used for monoculture of air-liquid differentiated airway epithelial cells. Others have used media used for the culture of immunocytes, which may not be optimal for the culture of structural cell types.

A discussion of experimental replicates when using PCLS also requires careful attention; some report experimental replicates as those data derived from multiple slices from a single lung donor. This approach fails to account for biological variation across multiple donors. Arguably, technical replicates can refer to serial slices cut from the same core/piece of lung tissue, or may refer to multiple slices derived from a single donor. A designation of biological replicates, however, should only be applied to data derived from separate, distinct donors and should not be published as experimental replicates if they are all derived from a single donor. Other investigators refer to a variant of PCLS in which bronchioles are embedded in agarose and thinly sliced [306–308]. This system is more akin to organ bath systems where bronchiole rings are cut and tethered to a myograph to measure force generation of airway smooth muscle. PCLS can measure an integrated response that incorporates parenchymal tethering of the airway and is more akin to an in situ environment in which airway luminal area is measured. Accordingly, the bronchiole slice model may not equate to PCLS and may serve to measure outcomes disparate from those measured with PCLS. In summary, standardization of protocols for the generation and use of PCLS will improve scientific rigor and reproducibility.

## Conclusions and future directions

PCLS provide a multifaceted platform in which studies of several different lung diseases can be studied. The mechanistic insights gained for a range of lung diseases provide novel targets for development of therapeutics that can be used in conjunction with current treatments, or may even serve as replacements to traditional therapeutic strategies. The PCLS platform offers a broadly applicable model for treatment of an array of lung diseases, with the platform providing a solid connection between translational science and clinical utility.

## Data Availability

No datasets were generated or analysed during the current study.

## Abbreviations

PCLS: Precision cut lung slices
hPCLS: Human precision cut lung slices
SARS-CoV2: Severe acute respiratory syndrome-related coronavirus
RV: Rhinovirus
RSV: Respiratory syncytial virus
LPS: Lipopolysaccharide
PH: Pulmonary hypertension
BPD: Bronchopulmonary dysplasia
IPF: Idiopathic pulmonary fibrosis
COPD: Chronic obstructive pulmonary disease
FEF25-75: Force expiratory flow between 25 and 75%
IgE: Immunoglobulin E
IL-13: Interleukin 13
β_2_AR: β_2_ adrenergic receptor
CS: Cigarette smoke
CSE: Cigarette smoke extract
PDE: Phosphodiesterase
PGES-1: Prostaglandin E synthase 1
5-HT: 5-hydroxtryptamine
LTD4: Leukotriene D4
siRNA: Small interfering ribonucleic acid
IFN γ: Interferon γ
TGFβ: Transforming growth factor β
