# Correction: Precision cut lung slices: an integrated ex vivo model for studying lung physiology, pharmacology, disease pathogenesis and drug discovery

**DOI:** 10.1186/s12931-024-03085-6

**Published:** 2025-01-27

**Authors:** Cynthia Koziol-White, Eric Gebski, Gaoyaun Cao, Reynold A. Panettieri Jr.

**Affiliations:** Rutgers Institute for Translational Medicine and Science, The State University of NJ, Rutgers, New Brunswick, NJ 08901 USA

**Correction to: Respiratory Research (2024) 25:231** 10.1186/s12931-024-02855-6

In the original publication of this article [1], the competing interests was missing from this article and should have read ‘Cynthia Koziol-White and Reynold A. Panettieri Jr. are Guest Editors for the *Human precision cut lung slices: an ex vivo platform for therapeutic target discovery and drug testing in lung disease* collection in which this article is published in’.

The original article has been updated.
